# Reported Energy Intake Accuracy Compared to Doubly Labeled Water and Usability of the Mobile Food Record among Community Dwelling Adults

**DOI:** 10.3390/nu9030312

**Published:** 2017-03-22

**Authors:** Carol J. Boushey, Melissa Spoden, Edward J. Delp, Fengqing Zhu, Marc Bosch, Ziad Ahmad, Yurii B. Shvetsov, James P. DeLany, Deborah A. Kerr

**Affiliations:** 1 Epidemiology Program, University of Hawaii Cancer Center, Honolulu, HI 96813, USA; yshvetso@cc.hawaii.edu; 2 Department for Structural Advancement and Quality Management in Health Care, Technical University Berlin, Berlin 10632, Germany; Melissa.spoden@googlemail.com; 3 School of Electrical and Computer Engineering, Purdue University, West Lafayette, IN 47907, USA; ace@ecn.purdue.edu (E.J.D.); zhu0@ecn.purdue.edu (F.Z.); 4 The Johns Hopkins University Applied Physics Laboratory, Laurel, MD 20723, USA; Marc.Bosch.Ruiz@jhuapl.edu; 5 Motorola Mobility LLC, Chicago, IL 60654, USA; ziad@motorola.com; 6 Department of Medicine, Division of Endocrinology and Metabolism, University of Pittsburgh, Pittsburgh, PA 15213, USA; jpd21@pitt.edu; 7 School of Public Health, Curtin University, Perth WA 6845, Australia; d.kerr@curtin.edu.au

**Keywords:** dietary assessment, mobile food record, image-based dietary assessment, doubly labeled water, adults

## Abstract

The mobile Food Record (mFR) is an image-based dietary assessment method for mobile devices. The study primary aim was to test the accuracy of the mFR by comparing reported energy intake (rEI) to total energy expenditure (TEE) using the doubly labeled water (DLW) method. Usability of the mFR was assessed by questionnaires before and after the study. Participants were 45 community dwelling men and women, 21–65 years. They were provided pack-out meals and snacks and encouraged to supplement with usual foods and beverages not provided. After being dosed with DLW, participants were instructed to record all eating occasions over a 7.5 days period using the mFR. Three trained analysts estimated rEI from the images sent to a secure server. rEI and TEE correlated significantly (Spearman correlation coefficient of 0.58, *p* < 0.0001). The mean percentage of underreporting below the lower 95% confidence interval of the ratio of rEI to TEE was 12% for men (standard deviation (SD) ± 11%) and 10% for women (SD ± 10%). The results demonstrate the accuracy of the mFR is comparable to traditional dietary records and other image-based methods. No systematic biases could be found. The mFR was received well by the participants and usability was rated as easy.

## 1. Introduction

Dietary data provide some of the most valuable insights into the occurrence of disease and subsequent approaches for mounting intervention programs for prevention. Due to the high daily and individual variance of diet, the accurate assessment of dietary intake is more challenging than the measurement of many other environmental exposures. Thus, dietary assessment methods need to continue to evolve to address these challenges [1,2,3]. The rapid uptake of mobile devices by the public offers a suitable platform for dietary assessment [4]. Two branches using images captured by mobile devices to estimate dietary intakes have evolved: image-based and image-assisted methods [5]. While image-assisted methods take images as a reference to adjust participants’ statements made in a 24-h dietary recall or recorded in a dietary record, the image-based methods use the captured images as the primary record of dietary intake [5]. A review of 13 studies that evaluated 10 image-assisted methods among adults aged 18 to 70 years indicated images enhance self-report made using traditional assessment methods by revealing unreported foods and misreporting of portion sizes [6]. Image-based methods integrating either smartphone cameras [7,8] or wearable cameras [9] have potential to provide valid estimates of energy intakes [6]. However, for both image-assisted and image-based methods underreporting can still occur if users miss taking an image or the images are not of sufficient quality [6]. Controlled feeding studies where true intake is known are ideal for validating short-term dietary assessment methods [10]. However, these methods are not practical for capturing intake for longer periods. Thus, biomarkers allow for testing in the community using established reference methods [11]. Doubly labeled water (DLW) which measures total energy expenditure (TEE) can translate to reported energy intake (rEI) under circumstances of energy balance, i.e., no significant weight loss or gain [12] and has been shown to provide an accurate measure under community dwelling conditions [13,14]. To date, there have been few validation studies conducted with either image-assisted or image-based methods [7,9,11]. In all of these studies, the sample size was small and detailed participant usability data was not included.

The mobile food record (mFR), an application designed specifically for assessment of dietary intake, uses the camera of a mobile device to capture food intake and to estimate energy and nutrient intake. After testing the mFR under controlled conditions to ascertain the theoretical functioning [3,4,15,16,17], the Food in Focus study examined the accuracy and usability of the mFR under community dwelling conditions among an adult study sample. The purpose of this study was to evaluate the accuracy of the mFR application as a tool for collecting rEI and its usability under real-life conditions among a diverse sample of healthy men and women aged between 21 and 65 years by using DLW as a recovery biomarker for TEE. The primary aim was to examine whether the dietary estimates for energy generated from 7 days of using the mFR would significantly (*p* < 0.05) correlate with TEE measured over the corresponding 7 days using DLW. A secondary aim was to determine whether the mean reporting of energy intake from 7 days of using the mFR compared to TEE measured over the corresponding 7 days using DLW would be 90% or greater.

## 2. Materials and Methods

### 2.1. Study Participants

Adults aged between 21 and 65 years residing in a Midwest rural county were recruited using flyers posted at community establishments, such as grocery stores, churches, and libraries. In addition, free media inserts to newspapers, church bulletins, and other publications were used. The county includes a large Land Grant university, thus recruitment methods outside the campus area were used to minimize recruitment of highly educated volunteers. Excluded were those who did not meet the age range, who practiced any extreme forms of exercise such as marathon training, and who travelled frequently outside the Midwest region (weekly or bi-monthly basis). An additional exclusion criterion was not having wireless internet access at home. The study methods described here were approved by the Purdue University Biomedical Institutional Review Board, West Lafayette, Indiana, USA on 03/11/2010 as protocol number 0707005629 and informed consent was obtained from all participants.

### 2.2. Study Design

**Figure 1:**
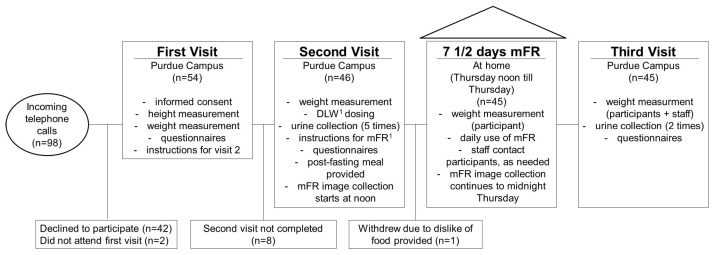
Study schedule and participant flow of the Food in Focus study. ^1^ Abbreviations: DLW, doubly labeled water; mFR, mobile food record.

The participants attended three visits, with a seven days study period between the second and third visit (Figure 1). At the first visit, the consent forms were confirmed. The participants completed questionnaires for characteristics, medical conditions and medications. Trained staff conducted measures of height, weight and bioelectrical impedance using standardized protocols. Participants were shown the menu of foods to be provided. Staff instructed the participants on the fasting conditions for the second visit. Upon completion of the first visit, participants were compensated $10 US.

On the second visit, staff weighed the participants. After baseline urine samples were collected, participants drank a mixture containing 1.8 g/kg total body water of 10% H_2_^18^O and 0.12 g/kg total body water of 99% ^2^H_2_O. Postdose urine samples were collected 4.5 and 6 h later, after urine voids were discarded at 1.5 and 3 h. During this time, the participants completed two physical activity questionnaires [18,19] and the Three Factor Eating Questionnaire [20]. After completing the questionnaires, each participant was provided with an iPhone 3GS with the mFR application preinstalled and a fiducial marker [21] (e.g., a checkerboard pattern of known shape, size, and color). The staff asked each participant about his/her usual eating times and accordingly installed three recording reminders. Participants were instructed to capture images prior to consuming any food using the “Before” button and to use the “After” button to capture the end of an eating occasion whether everything was eaten or not. After practicing with the mFR using plastic food replicas, a questionnaire capturing their initial opinions on the usability of the mFR was completed [3,15]. The participants were asked to weigh themselves each day during the remaining 7.5 days of the study using a LifeSource Pro-Fit Precision scale which was provided along with instructions for weighing themselves at home. A Daily Weight Record Booklet and a Things to Remember sheet were provided. After the 8 h fasting period for the DLW dosing, a complimentary meal was provided around 12 noon, which presented an opportunity to theoretically and practically train the participants in recording their eating occasions with the mFR. From this point forward, the participants captured images of every eating occasion up to the midnight prior to the visit 8 days later, resulting in a total of 7.5 days of capturing images with the mFR. Time stamps on the images and final confirmation with the participants allowed a sufficient level of accuracy for confirming this length of use.

Food pack-outs for the remainder of day one and the next two days of the study were distributed. Beyond the foods, the pack-outs included plates, clear glasses and a grey placemat. In the evening of the second total food provision day, the participant returned all uneaten foods from the previous days and picked up the foods for the next three days. The same procedure took place for returning the foods followed by picking up foods for the next two days. The uneaten foods of the last two days were returned on the final third visit. The returned portions were weighed and recorded for each participant. Providing known foods and amounts supported the objective of being able to identify the foods consumed and their amounts as further described in the sections below.

The participants were instructed to eat the foods as they normally would, e.g., they did not have to eat all the foods which were provided, they could eat dinner food items for breakfast. Furthermore, the participants were encouraged to supplement the provided foods with other foods and take images of these foods and beverages as well. For the completion of the second visit, participants received $20 US.

The study staff monitored the incoming images online and texted or emailed the participants, as needed, to make suggestions for improving image quality or if time gaps occurred between images. To monitor time gaps, a program running on the server would generate messages for the staff regarding the receipt of images using the study participant’s usual eating patterns to inform content of the message. The format of the message was “The server has not detected any activity from user #### over the last ## hours”. For each of the seven full study days, the participants received $10 US. At the third visit, the participants were weighed; the two final urine samples collected and two questionnaires assessing the usability of the mFR completed. For this last visit, the participants received $45 US.

### 2.3. Estimating Energy Content for the Food Pack-Outs

The 61 beverages, foods, and condiments provided in the pack-outs were selected to represent usual food items as informed by frequently consumed foods from the National Health and Nutrition Examination Survey (NHANES) (Table A1). The foods were distributed to fit the participants’ energy needs. The estimated energy requirement (EER) for each individual was estimated using the recommendations of the Institute of Medicine, Food and Nutrition Board for adults ages 19 years and older [22]. All items were pre-weighed and the pack-outs were prepared for energy levels of 2000, 2500, 3000, and 3500 kcal/day. Once the EER was computed, the pack-out just above an individual’s EER was assigned, e.g., a person with a 2300 EER would receive the 2500 pack-out. One participant had an EER above 3500 kcal, thus this pack-out was supplemented with additional foods.

### 2.4. Description of the mFR

**Figure 2:**
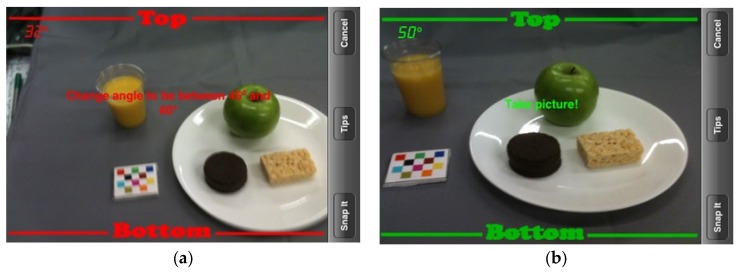
Image capturing with interchangeable color border indicating the correct angle. (**a**) The incorrect angle displays as red and (**b**) the correct angle displays as green.

**Figure 3:**
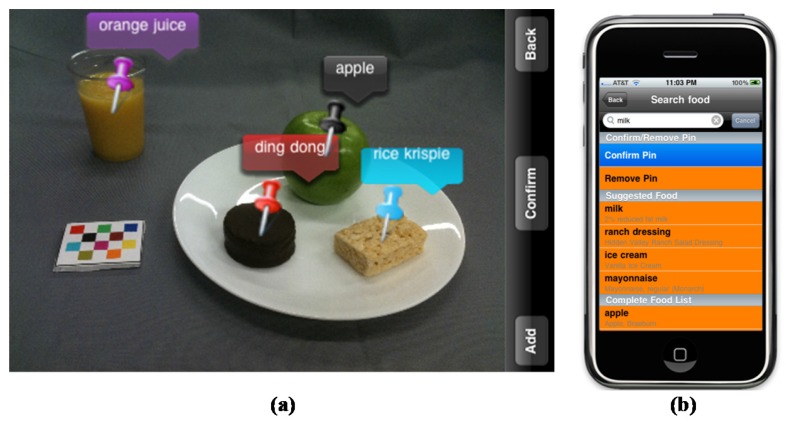
(**a**) This stylized image shows the review and confirmation process. Foods are identified with colored pins with matching colored labels; (**b**) When a pin is touched, the application displays the “confirm/remove pin” screen. For each food, four suggested foods are listed with the first food being the food that the classifier assessed as being the most likely food match. If the exact food is not listed in the top 4, the user can access the “complete food list”.

The mFR is an application for mobile devices to capture foods and beverages. The application is based on one of the technology assisted dietary assessment (TADA) protocols [4,21,23]. Crucial for the image analysis is the inclusion of the fiducial marker (FM) in the image [4,24,25]. The dimensions and color markings of the FM are known and used as a reference for the spatial and color calibration of the camera. The FM enables the identification of the foods and beverages as well as the portion-size estimation [25]. The image analysis depends on the angle from which the image is taken. On the screen, two interchangeable color borders, i.e., red or green, signal the user the angle to take the image (Figure 2). The accepted images get automatically uploaded to a central server when connectivity is available. The methods for the automatic food segmentation and identification have been described previously [24]. After the automated identification, the images are returned to the user for review and confirmation. Using the mFR application, the user is prompted to confirm or change the food identification displayed by labeled pins on the food items (Figure 3a). The system presents the user four Suggested Foods, beyond these the user is free to search for other foods in the Complete Food List (Figure 3b). Once confirmed the image with the confirmed pins is automatically sent to the server and disappears from the application. Participants were recommended to complete this process at the end of each day.

In general, community dwelling studies comparing dietary intake with DLW do not provide food pack-outs [26,27,28]. This represented a unique component of the study which was testing the automatic identification of foods and amounts. The engineering process of automatically identifying foods and their amounts from images is referred to as the “automated classifier”. Prior to enrolling participants in the Food in Focus study, the automated classifier was trained on the foods and beverages provided in the pack-outs. The images from the study participants informed the automated classifier under community dwelling conditions. The results and progress of this aspect of the study are published elsewhere [21,23,24].

### 2.5. Total Energy Expenditure

TEE was assessed using DLW as described by DeLany et al. [29]. The rate of CO_2_ production was calculated [30], and TEE was derived by multiplying by the energy equivalent of a respiratory quotient of 0.86.

### 2.6. Energy Intake

For this analysis, all before and after images taken by the participants were reviewed by three trained analysts, identifying and estimating all food items in the images according to a standardized protocol which included information about the pack-out menus and confirmation of foods and beverages not provided in the pack-outs. In the case of lacking consensus an adjudicator identified the food code and portion size. Based on these food codes and portion sizes, the reported energy intake (rEI) was estimated using the United States Department of Agriculture (USDA) Food and Nutrient Database for Dietary Studies (FNDDS) version 3.0.

The difference between the distributed and returned food portions was used to estimate the presumed energy intake (pEI) referencing to the energy intake derived solely from the provided foods. All energy data were normally distributed (Kolmogorov-Smirnov test, TEE = 0.121; rEI = 0.179; pEi = 0.106), therefore no transformation was needed. Energy intakes, as mean kcal/day, were compared by sex and method (TEE, rEI, pEI), using a paired t-test. The correlation of TEE and rEI was assessed by the Spearman correlation coefficient.

### 2.7. Identification/Quantifying of Misreporting

For the identification of misreporters the methods of Black and Cole were used [31]. In the case of accurate reporting the ratio of rEI to TEE was assumed to be 1. rEI values falling above or below the 95% confidence intervals (CI) of the ratio indicated under- or overreporting, respectively. The formula for calculating the 95% CI provided by Black and Cole was used [31]. For the within-subject coefficient of variation for TEE, needed for the calculation of the 95% CI, the value of 1.8% from the OPEN-study was used [32]. The number of under- and overreporters was determined by sex, body mass index (BMI) category, age category, rEI, and TEE. Additionally, Bland–Altman analysis was performed to determine systematic bias introduced by the amount of energy intake [33].

### 2.8. Statistical Methods

All statistical analyses were performed with IBM^®^ Corporation Released 2015. IBM SPSS Statistics for Windows, Version 23.0. Armonk, NY, USA: IBM^®^ Corporation. Descriptive statistics, such as means, standard deviations (SD) and percentages, stratified by sex were computed for the study sample characteristics. Answers to the questionnaires on usability [3,4,17], assessed before and after using the mFR, were analyzed as counts and percentages and Wilcoxon Signed Ranks test was used for the comparison of the answers before and after the study period. At the final visit, participants were asked an open question about length of time willing to use the mFR in days, weeks, or months. Responses were a single length of time or a span of time, in which case the shortest span of time was used. Overweight and obesity were categorized using the guidelines published by the National Institutes of Health [34].

## 3. Results

### 3.1. Characteristics of the Study Sample

From the 98 individuals screened for eligibility by telephone, 54 individuals completed the first visit and 46 the second visit. The reasons for non-participation and discontinuing after the first visit was the time commitment for the dosing of the DLW (*n* = 8) or not liking the foods provided at the second visit (*n* = 1) (Figure 1). Therefore, data were collected from 45 adults (15 men, 30 women) between 21 and 63 years of age (Table 1). The mean BMI was 26 kg/m^2^ (SD = 6) and the mean age was 33 years (SD = 12). The participants were predominantly (73%) non-Hispanic White (Table 1) and were predominantly considered active [35]. Among all participants, the mean weight measured by staff on the first day using the mFR did not significantly differ from the weight measured by staff on the last day of the study (paired *t*-test, *p =* 0.694; mean difference −0.06 kg). The percent mean weight change was −0.01% for the total sample (results not shown), with +0.3% for men and −0.2% for women. Forty participants provided images for 7.5 days. One participant sent images for 2.5 days, one participant 5.5 days, two participants 6.5 and, one participant for 7.0 days of the study. The mean was 7.3 days (SD = 0.8).

### 3.2. Energy Intake

**Figure 4:**
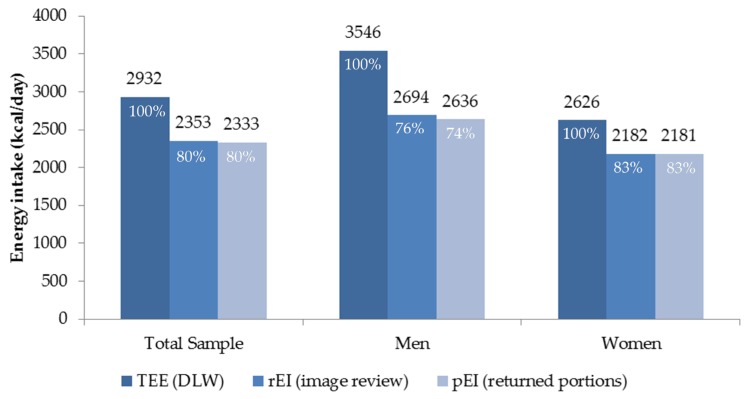
Comparison of mean total energy expenditure (TEE) based on doubly labeled water (DLW), reported energy intake (rEI) using images from the mobile food record, and presumed energy intake (pEI) based on returned preweighed servings of food over 7 ½ days by total sample (*n =* 45) and sex (men = 15 and women = 30).

Mean values were 2932 kcal/day for TEE and 2353 kcal/day for rEI with a resulting difference of 579 kcal/day (Figure 4). Stratified by sex, the difference between TEE and rEI was greater for men (852 kcal/day) and smaller for women (444 kcal/day). The difference between rEI and pEI was 20 kcal/day among all participants, i.e., 58 kcal/day among men and 1 kcal/day among women (Figure 4). A paired *t*-test showed a significant difference between the mean daily TEE and rEI (*p <* 0.0001; mean difference 580 kcal). Median TEE measured by DLW was 2846 kcal and median rEI was 2255 kcal. The Spearman coefficient indicated a moderate statistically significant correlation of 0.58 (*p <* 0.0001).

In the images of foods consumed and not provided in the pack-outs, the primary items were sugar sweetened beverages, coffee, tea, and alcohol. A list of the foods recorded and enumerated in the images that were not in the pack-outs is presented in Table A2.

### 3.3. Energy Misreporting

All participants with values of the ratio rEI:TEE within the 95% CI (0.8–1.2) were classified as accurate reporters, participants with rEI:TEE values below or above the 95% CI as underreporters or overreporters, respectively (Table 2). Accurate reporters comprised 44% of the sample and 2% were overreporters. Across the entire sample, 53% of the participants were classified as underreporters, and the mean underreporting was 563 kcal per day less than TEE measured by DLW. Underreporters showed a mean difference of 1000 kcal/day between rEI (2138 kcal/day) and TEE (3138 kcal/day). The mean difference for accurate reporters was 158 kcal between rEI (2515 kcal/day) and TEE (2673 kcal/day). Men were more likely to be classified as underreporters than women, 73% and 43%, respectively. No clear trend in rEI:TEE emerged either for age or BMI. A larger non-statistically significant proportion of obese participants underreported (60%) than reported accurately (30%) (Table 2).

**Figure 5:**
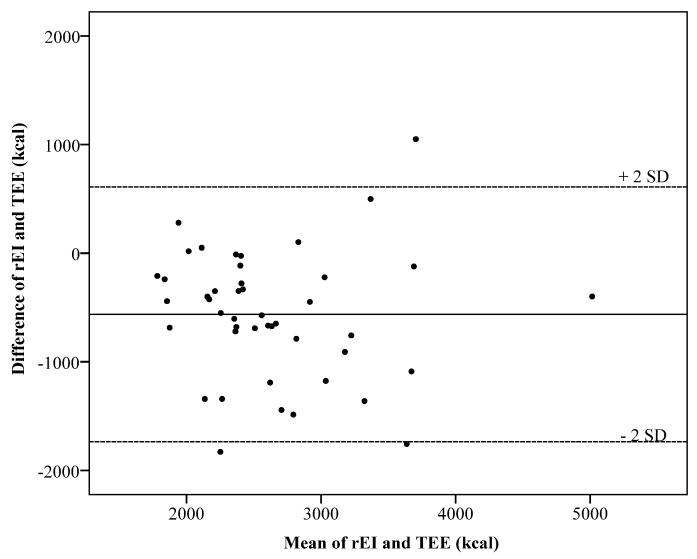
Bland–Altman plots showing the difference in kcal between the total energy expenditure (TEE) measured using double labeled water (DLW) and reported energy intake (rEI). (SD, standard deviation).

The mean percentage of underreporting was 12% for men (SD ± 11%) and 10% for women (SD ± 10%). The Bland–Altman plot does not indicate a systematic bias with an increasing energy intake level (kcal) (Figure 5). The reporting accuracy was consistent over all energy intake levels.

The horizontal axis represents the mean of rEI and TEE in kcal. The vertical axis represents the difference between rEI and TEE in kcal. The solid line represents the mean difference of −563 kcal. The two dashed lines represent the limits of agreement, defined as the mean difference plus and minus 2 times the standard deviation of the difference.

### 3.4. Usability

Perceptions of using the mFR were assessed before and after the 7.5 study days (Table 3). The majority, 71%, agreed or strongly agreed to the statement: ‘Remembering to take an image before meals would be easy’ before the start of the study. After the week of using the mFR, the agreement rate rose to 100% (*p <* 0.0001). A similar pattern was observed for the statement on remembering to take the images after meals, with 71% agreeing or strongly agreeing before the study and 76% (*p =* 0.646) after the study. For remembering to take images of snacks, the agreement before the study was 38% and 47% for images before and after eating, respectively. After the study, the agreement climbed to 80% (*p <* 0.0001) for before images and 64% (*p =* 0.065) for after images (Table 3). The perception of being easy to carry a credit card sized fiducial marker remained the same 91% to 93% (*p =* 0.827), whereas the proportion of individuals thinking the use of the fiducial marker was easy increased from 87% to 96% (*p =* 0.670).

Responses to questions completed after using the mFR for 7.5 days are summarized in Table 4. The majority of the responses were positive about the experience of using active image capture of foods eaten, e.g., 84% agreed being comfortable using the application and 96% were confident that the information collected by the TADA iPhone application would only be seen by researchers and not used against the participant.

In an open-ended question, the participants were asked what they liked the most about the mFR. Examples of frequent responses are the mFR is easy to use, labeling of the foods was fun or enjoyable, and it helped them to keep track of what they ate or to restrict their food intake. This latter response was consistent with the response to the question, “Did using the TADA iPhone application make you behave differently than if you didn’t have the TADA iPhone application?” with 69% saying yes. When asked what they liked the least about the mFR, often mentioned was the labeling of foods as too time consuming or the accuracy of the automatically set labels as too low. A number of participants found the connection to the server too slow or it took too long for them to get the images back for reviewing. The set up for an eating occasion using the placemat etc. was too cumbersome or too hard to remember. Many admitted when they were either in a hurry, in a public place or snacking, these situations made them think about not taking an image.

When asked how long they would be willing to use the mFR in days, weeks, or months, all participants indicated a time period within the range of 3 days (*n =* 1) to 6 months (*n =* 1). For the number of days, the range was 3–90 days and the mode was 30 days (*n =* 20). The number of weeks ranged from 0 (*n =* 1) to 30 (*n =* 1) with the mode being 4 weeks (*n =* 19). For months of use, the most frequent response was 1 month (*n =* 20).

## 4. Discussion

The dietary estimates for energy generated from 7.5 days of using the mFR were significantly (Spearman correlation coefficient of 0.58, *p <* 0.0001) correlated with TEE measured over the same time period using DLW. The mean rEI:TEE ratio was 81% for the total sample (men 76%; women 84%), resulting in a mean underreporting among men of 24% among men and 16% among women.

Three prior studies compared TEE to rEI using traditional 7 days dietary records among ideal study samples. McClung et al. [36] studied active young military men (*n =* 24) and women (*n =* 2) with a mean age of 23 years. The participants used a Personal Digital Assistant to enter real-time dietary intake. The rEI:TEE ratio among this sample of active military duty adults was 0.92. In another study, 838 women were screened for psychosocial and health issues and motivation [37]. After screening, 22 women with a mean age of 30 years met the eligibility criteria to participate in the study of 7 days of dietary records and DLW dosing. Data were available for analysis from 20 members of the motivated sample of women. The resulting rEI:TEE ratio among this group was 0.94. For the third study, ten dietitians were recruited to complete 7 days of dietary records for comparison to DLW [27]. The average age of this sample of women was 36 years. For comparison, non-dietitian women with a mean age of 33 years were recruited. This non-dietitian group had a rEI:TEE ratio of 0.81. The rEI for the dietitians was not significantly different from TEE; whereas the results for the non-dietitians were significantly different. These authors hypothesized that the dietitian’s professional experience with food likely contributed to the energy intakes not being significantly lower than the energy expenditure. In comparison to the results from the selective individuals in these studies [27,36,37], the rEI:TEE ratio of 0.84 for women in the Food in Focus study suggests a relatively high accuracy.

Using recruitment methods similar to the Food in Focus study, Barnard et al. [28] recruited men and women between 22–59 years with a BMI of 19 to 33 kg/m^2^. The final sample was smaller, i.e., 7 women and 7 men; otherwise the participants’ characteristics aligned closely to the Food in Focus study. The results of Barnard et al. [28] were similar to the Food in Focus study with regard to no significant relationship between traditional 7 days dietary records and misreporting and BMI. The rEI:TEE for the participants in the Barnard et al. [28] was 0.74 for men compared to 0.76 for men in the Food in Focus study. For women, this same comparison is 0.54 compared to 0.84 in the Food in Focus study. In the Barnard et al. study, higher misreporting was associated with a higher number of dinner foods, younger age, a wider range of foods, and being female. The better results from the Food in Focus study could be due to the lower recording burden associated with the mFR as only one image is needed to capture one food or many foods at any one eating occasion. Unlike Barnard et al. findings, no association with age was found in the Food in Focus study and research would suggest that younger individuals are more likely to embrace using mobile telephones over hand writing [17]. Further, in the case of using the mFR, women recorded more accurately than men; the opposite of results of Barnard et al.

Trabulsi and Schoeller (2001) analysed the reporting accuracy in 30 studies with at least 10 participants comparing TEE to rEI using dietary records [38]. Five studies in this review, involved collection of dietary records over 7 days [39,40,41,42,43]. The level of misreporting ranged from a high of 37% among obese men [40] to a low of 18% among normal weight women [42]. These results would support the mFR as a more accurate method. Of these 5 studies, only one study [43] reported a correlation coefficient result (*r* = 0.46, <0.01) which was lower than the Spearman correlation coefficient in the Food in Focus study.

Several validation studies of image-assisted methods have been published [5]. Using a DLW protocol, Pettitt et al. [9] found underreporting of 34% when using a micro-camera worn on the ear to assist a 2 days dietary record among 6 study volunteers. The use of the data secured through the micro-camera reduced the underreporting to a mean of 30%. The participants reported not being comfortable wearing the device in public and that it would affect their activities. These reactions contrast sharply to the positive usability and level of comfort responses received for the mFR in the current study where acceptability was high, with 73% of participants willing to participate in another study using the mFR. The SenseCam (Microsoft Research, Redmond, WA, USA) can be used to take images automatically during eating occasions to assist with reporting for a 24-h dietary recall [11]. After complementing the interview results with the captured images, the underreporting was reduced to 9% for the 20 men and 7% for 20 women completing the study. Of interest, participants using the SenseCam did not have the same levels of discomfort expressed by participants using micro-camera described above. The Nutricam Dietary Assessment Method (NuDAM) is an image-based dietary record that also uses voice recording and follow-up telephone calls for detail confirmation. Ten adults with type 2 diabetes participated in a protocol comparing the results of NuDAM to a traditional dietary record and TEE assessed with DLW. The results from NuDAM were equivalent to the written dietary record. Each showed underreporting of 24% among the 6 men and 4 women completing the protocol [7]. NuDAM includes a series of activities that are complex for the user. Given that the system is being designed for individuals diagnosed with diabetes, the effort involved is likely seen as less burdensome, as the individuals preferred NuDAM over the written dietary record. Whereas many of the Food in Focus participants liked confirming the pins on the images in the mFR application, which is similar to one of the steps in NuDAM, other Food in Focus participants wanted this to go faster. The mFR uses as few steps as possible in order to maintain cooperation, which appears to be reflected in the high proportion of participants completing recording for 7.5 days.

In the Food in Focus study, men were more likely to be underreporters than women. Previous studies have not found a consistent pattern of sex influencing underreporting [32,44]. An association of underreporting and overweight was reported by earlier studies using 24-h dietary recalls [13,32,44], these results could not be replicated in this study, which implies that no bias of reporting associated with BMI is introduced by the mFR. Also contrary to previous studies, no systematic bias regarding underreporting and energy intake levels could be seen in the Food in Focus data [32,44]. However, replication in a large sample is necessary to confirm the assumption made on the lack of a systematic reporting bias. The provided menu of the present study may have affected the eating behavior and especially the energy intake of the participants. However, there is no reason to suspect that the accuracy of the mFR would be significantly different if no foods would have been provided. Previous studies have indicated daily biases in food consumption throughout the week. Haines et al. found that from Friday until Sunday, adults aged between 19 and 50 years increased their energy intake about 115 kcal/day [45]. Future analysis aiming to find the sufficient number of recording days for accurate habitual intake estimation should take differences in energy intake throughout the week into account.

Errors on the individual level could be introduced by the DLW method itself. A validation study stated accuracy of 1.3% ± 8.9% SD between TEE measured by DLW and a metabolic chamber, but on the individual level the errors ranged from −17.7% to +12.5% [46]. Moreover, due to the study design, the computation of TEE was based on a mean of 8.5 days, whereas rEI was estimated using individual numbers of recording days. To be able to distinguish between underreporting and true undereating, TEE and weight change should be taken into account. Due to the study duration of 7.5 days, the weekly within-subject variation in weight change might introduce more random error than actual weight change induced by a reduction of body mass [32].

Misreporting of dietary intake with food records is well recognized [47]. Participants in the current study may have misreported by not taking images of some additional food or beverages. Reactivity bias may occur when an individual changes their behavior due to awareness of being measured. A common finding of food records is an increased awareness of diet and behavior changes amongst the participants [48,49], which cannot be ruled out in the current study. In response to an open question on what the participants liked the most about the mFR, 13% mentioned that it helped them to keep track of what they ate or to restrict their food intake. To the question whether the mFR made them behave differently, 69% answered yes. Despite these comments, no large weight changes were observed. However, over longer term, these changes in behavior should be prospectively explored, taking weight change into account and using qualitative questionnaires on the details of behavior change.

Preferences for image-based methods over traditional dietary assessment methods were captured in previous studies [50,51,52]. Using the mFR was considered to be easy by 82% and only 4% stated that the mFR was not enjoyable to use. 73% stated that they would be willing to use the mFR for more than 7 days, with 60% stating a time period ranging between 1 to 6 months. On the question what they liked the least about the mFR, 27% mentioned the labeling of foods as too time consuming or the accuracy of the automatically set labels as too low. 18% found the connection to the server too slow or that it took too long to get the image for reviewing back. Improving non-technology based methods has been challenging in the past. In contrast, the technology concerns expressed by the mFR users, i.e., better connectivity speed for image transfer, faster devices to speed up labeling of foods; have been addressed through the continuous progress made to advance technology. Concurrently, the feedback received from the participants has also been addressed through improved programming. Most of the concerns expressed by participants have been addressed or can be addressed as part of the evidence based process [4]. Unlike systems used in the past, using flexible applications have distinct advantages as issues found during use can be immediately addressed and this level of adaptive response will only continue as systems advance.

For automated identification, capturing the color and texture characteristics of foods is essential. The colored fiducial marker with its standardized size and features plays a crucial role in the automation. Consistent with past studies [3,4], individuals found the fiducial marker easy to use and even identified it as fun.

Unresolved is when and how the underreporting (under picture taking) took place and which foods were missed. The foods supplied met the study participants’ energy needs. The energy difference of the provided foods from the returned foods matched almost exactly the energy content of the foods and beverages assessed in the images. Despite this observation, the foods and beverages in the images included multiple foods that were not supplied by the study. The majority of the foods not provided and visible in the images were energy rich foods, e.g., alcohol, sugar sweetened beverages (Table A2). Consumption of these items on top of the provided food would be consistent with energy intakes being higher than energy balance. Only one individual was above the expected TEE level. The foods included in the images and not provided are often considered socially unacceptable; as such their inclusion in the images would be unexpected. As an explanation, the possibility exists that the study participants may have shared food with friends/family or, consistent with previous studies, participants did not capture all of the food eaten. And since the food was eaten, it could not be returned. As noted by Hebert [53], future studies need to explore responses using different assessment methods and attempt to assess psychological predispositions influencing motivation, expectation, and self-efficacy.

Strengths of this study include DLW as the reference method to validate rEI, the image review process by three trained analysts as well as the combination of qualitative and quantitative methods. Limitations include the small sample size which restricts the analysis of subgroups and therefore the detailed exploration of possible participant related biases introduced by the mFR. The provided menus may have altered usual eating habits of the participants and could have incented participants to eat more. However, this study serves as validation of rEI measures and justifies the implementation of a study recruiting a larger sample.

## 5. Conclusions

This study amongst 45 community dwelling adults aged between 21 and 65 years assessed the validity of the mFR compared to TEE measured by DLW. The results of lower underreporting demonstrate the accuracy of the mFR when compared to traditional food records and other image-based food records. No systematic biases regarding reporting could be found. This places the mFR in a superior position to other assessment methods. The mFR was well received by the participants and usability was rated as easy, unlike more traditional methods. Some modifications regarding the food labeling and the review process would be helpful to make recording more manageable throughout daily routines.

In the future, the mFR needs to be tested in a larger sample without a provided menu over an extended time period. Such a study would allow confirmation of weight and behavior changes. Furthermore, the analysis of different subgroups would provide more insight on possible participant related reporting biases.

## Figures and Tables

**Table 1:** Characteristics of the Food in Focus study sample (*n* = 45).

Characteristics	Men	Women
	*n =* 15	*n =* 30
BMI ^1^ categories (NIH ^1^)	*n*	
Underweight	0	2
Normal weight	7	12
Overweight	4	10
Obese	4	6
Hispanic or Latino	2	2
Black	0	2
White	13	20
Asian	2	7
Another Race	0	1
Active ^2^	10	18
Insufficiently active	5	12
	Mean ± SD ^1^	
Age (years)	32 ± 9	33 ± 13
BMI (kg/m^2^)	27 ± 5	26 ± 7
Height (cm)	180 ± 7	166 ± 6
Weight (kg)	87 ± 20	73 ± 19
Weight Change (%)	0.3 ± 0.6	0.2 ± 1
Completed days of record	7	6.7
Reported energy intake (rEI) (kcal/day)	2694 ± 794	2182 ± 577
Presumed energy intake (pEI) (kcal/day)	2636 ± 692	2181 ± 517
TEE ^1^ (kcal/day)	3546 ± 681	2626 ± 492

^1^ Abbreviations: BMI, body mass index; NIH, National Institutes of Health; SD, standard deviation; TEE, total energy expenditure; ^2^ Godin-Shephard Leisure-Time Physical Activity Questionnaire classification using only moderate and strenuous scores [35].

**Table 2:** Levels of reporting accuracy by participants’ characteristics.

Characteristics	Underreporter	Accurate Reporter	Overreporter
Variable (*n*)	*n* (%)	*n* (%)	*n* (%)
Total (45)	24 (53)	20 (44)	1 (2)
Male (15)	11 (73)	4 (37)	
Female (30)	13 (43)	16 (53)	1 (3)
Body mass index category			
Underweight (2)		2 (100)	
Normal weight (19)	11 (58)	8 (42)	
Overweight (14)	7 (50)	7 (50)	
Obese (10)	6 (60)	3 (30)	1 (10)
Age (years)			
20–29.9 (28)	15 (54)	12 (43)	1 (3)
30–39.9 (6)	4 (67)	2 (33)	
≥40 (11)	5 (46)	6 (55)	
	Mean ± SD	Mean ± SD	
rEI ^1^ (kcal/day)	2138 ± 471	2,515 ± 756	4230
TEE ^1^ (kcal/day)	3138 ± 596	2,673 ± 774	3180
rEI:TEE	0.68 ± 0.10	0.94 ± 0.10	1.33

^1^ Abbreviations: rEI, reported energy intake; TEE, total energy expenditure.

**Table 3:** Perception of usability of the mobile food record (mFR) before and after 7.5 days of recording among adults (*n =* 45).

Questions Asked before and after Using the Technology Assisted Dietary Assessment (TADA) mFR (Before Phrase/After Phrase)	Before 7.5 Study Days ^1^ *n* (%) of 45	After 7.5 Study Days ^1^ *n* (%) of 45
Remembering to take an image BEFORE MEALS would be easy/was easy.	32 (71)	45 (100) ^2^
Remembering to take an image AFTER MEALS would be easy/was easy.	32 (71)	34 (76)
Remembering to take an image BEFORE SNACKS would be easy/was easy.	17 (38)	36 (80) ^2^
Remembering to take an image AFTER SNACKS would be easy/was easy.	21 (47)	29 (64)
I think it would be /I thought it was easy to carry a CREDIT CARD sized fiducial marker.	41 (91)	42 (93)
I think it would be/I thought it was easy to use a CREDIT CARD sized fiducial marker	39 (87)	43 (96)

^1^ Only those selecting “strongly agree” or “agree” are shown here, the remainder selected “neither agree or disagree,” “disagree,” or “strongly disagree”; ^2^ *p* < 0.0001, comparing before to after 7.5 study days.

**Table 4:** Responses from adults (*n =* 45) completing the usability questionnaire after using the mobile food record (mFR) application for 7.5 days.

Statements and a Question Regarding Use of the Technology Assisted Dietary Assessment (TADA) mFR Application	Responses, *n* (%)		
	Strongly Agree or Agree	Neither Agree or Disagree	Disagree or Strongly Disagree
I found it easy to include the fiducial marker in the picture of my meals.	38 (84)	4 (9)	3 (7)
I found it easy to include the fiducial marker in the picture of my snacks.	40 (89)	3 (7)	2 (4)
The screens were easy to read.	45 (100)	0	0
The TADA application on the iPhone was easy to use.	37 (82)	5 (11)	3 (7)
The directions about when to take an image of my meals and snacks were easy to follow.	43 (96)	2 (4)	0
The TADA iPhone interfered with my daily activities.	15 (33)	19 (42)	11 (24)
The TADA iPhone interfered with my social interactions.	15 (33)	13 (29)	17 (38)
I would like to participate in another study using the TADA iPhone application.	33 (73)	8 (18)	4 (9)
The directions about how to use the TADA iPhone application were easy to follow.	44 (98)	1 (2)	0
Overall, the TADA iPhone application was a nuisance to use.	5 (11)	20 (44)	20 (45)
Overall, the TADA iPhone application was enjoyable to use.	22 (49)	21 (47)	2 (4)
The extra cords helped keep the TADA iPhone charged at all times.	35 (78)	8 (18)	2 (4)
It was easy to use the TADA iPhone application when I was away from home.	26 (58)	7 (16)	12 (27)
It was easy to carry two phones.	25 (56)	6 (13)	14 (31)
More instructions about how to use the TADA iPhone application would have been helpful.	8 (18)	14 (31)	23 (51)
Understanding the purpose of the TADA iPhone application motivated me to use it.	28 (62)	15 (33)	2 (4)
I feel confident that the information collected by the TADA iPhone application will only be seen by researchers and not used against me.	43 (96)	1 (2)	1 (2)
	Never or almost never	Sometimes	Fairly or very often
I had problems using the TADA iPhone application.	23 (51)	16 (36)	6 (13)
	Extremely or mostly comfortable	Somewhat comfortable	Not too comfortable or not comfortable at all
Did you feel comfortable using the TADA iPhone application?	38 (84)	6 (13)	1 (2)

## Appendix A

**Table A1:** List of 61 beverages/foods/condiments provided to Food in Focus participants.

Bread, Bagel, Plain	Fruit, Orange, Clementine	Pretzels
Bread, English Muffin	Fruit, Orange, Navel	Pudding, Chocolate
Bread, Texas Toast	Fruit, Pear, Bartlett, fresh	Rice Krispie Treat
Bread, Whole Wheat	Fruit, Strawberries, fresh	Sandwich, Ham and Cheese
Cereal, Wheaties	Fruit, Watermelon	Sandwich, Turkey Wrap
Cheese, Cream, Plain, condiment pkt	Granola Bar	Sausage, Turkey
Cheese, Mozzarella Sticks	Ice Cream, Vanilla Sandwich	Snickers Bar
Cookies, Chocolate Chip	Jelly, Strawberry, condiment pkt	Soup, Chicken Noodle
Cookies, Snicker Doodle	Juice, Orange	Syrup, Maple, condiment pkt
Crackers, Goldfish	Lasagna, Lean Cuisine	Turkey Tettrazini, Stouffer’s
Crackers, Saltines	Lemonade	Veg, Broccoli w/cheese sauce
Ding Dong, Chocolate Cake Roll	Margarine, condiment pkt	Veg, Carrots, baby
Dip, Ranch Dressing, condiment pkt	Mayonnaise, condiment pkt	Veg, Celery, sticks
Doritos Chips	Meatloaf, Stouffer’s	Veg, Mixed/Lettuce Salad
Dressing, Fat Free Italian, condiment pkt	Milk	Veg, Peas
Dressing, Ranch, condiment pkt	Muffins, Mini	Veg, Potatoes, steamed
Frozen Fruit Bar	Mustard, condiment pkt	Veg, Tomatoes, Grape
Fruit, Apple, red	Pancakes	Yogurt, Mixed Berry
Fruit, Banana	Peanut Butter, condiment pkt	Yogurt, Strawberry
Fruit, Cocktail	Pizza, Stouffer’s French Bread	
Fruit, Grapes	Potato Chips	

**Table A2:** List of beverages and foods not provided in Food in Focus pack-outs and appeared in eating occasion images.

Beer	Margarita
Coffee	Marshmallow
Coffee latte (unsweetened)	Mellow Yellow/Mountain Dew/Orange Soda
Coke Zero/Diet Coke	Powerade
Coke/Pepsi	Red Wine
Diet Mountain Dew	Reese’s Cup
Dr. Pepper	Sprite/7-Up
Fruit punch	Tea (sweetened or flavored)
Gatorade	Tea (unsweetened)
Ginger Ale	Tortilla chips, Tostitos tortilla chips
Hawaiian Punch/Cran-Apple Juice/Hi-C	Water
Lemonade, Minute Maid Lemonade	
